# Investigative study on the antimicrobial activity and effects of virucidal hand antiseptic on skin

**DOI:** 10.1186/1753-6561-5-S6-P268

**Published:** 2011-06-29

**Authors:** E Noguchi, N Koval, T Andoh, M Ryu, M Yamamoto

**Affiliations:** 1 Biochemical laboratory, Saraya Co. Ltd., Osaka, Japan; 2 Graduate School of Medicine and Pharmaceutical Sciences, University of Toyama, Toyama, Japan

## Introduction / objectives

The efficacy and skin compatibility of virucidal hand rub was investigated.

## Methods

The efficacy of several commercially available virucidal hand antiseptics against FCV was compared by quantitative suspension test. The efficacy of an acidic formulation VH (Alsoft V, Saraya Co., Ltd.) against various types of microorganisms was further investigated according to international standards such as ASTM or EN.

The influence on mouse skin after repeated use of VH was also examined. After 5 days of use simulated in clinical practical condition, physiological properties were measured. In addition, a test on skin irritation was performed electrophysiologically.

## Results

Among the antiseptics tested, the acidic formulations showed efficacy against FCV even in short contact time of 15 seconds. VH also exhibited excellent efficacy against all the microorganisms tested.

After repeated use of VH, no significant difference in the gross appearance, TEWL, SC hydration and the number of epidermal fovea of mouse skin were observed. Skin bacterial count was lower than reference products. Regarding skin irritation, VH showed no peripheral nerve stimulation.

## Conclusion

This study shows that some commercially available virucidal hand antiseptics could not demonstrate sufficient efficacy against FCV in 30 seconds. In order to achieve the efficacy in short contact time, it is considered critical to formulate alcohol solution with acidic pH. Also, our formulation VH indicated the excellent efficacy against non-enveloped viruses, and against broad spectrum of microorganisms. With its effects on skin, it is suggested that VH has favorable moisturizing and sustainable antibacterial effect.

## Disclosure of interest

None declared.

